# Tuberculosis

**Published:** 1902-06

**Authors:** H. McHatton

**Affiliations:** Macon, Ga.


### TUBERCULOSIS.*

BY H. McHATTON, M.D.,

MACON, GA.

There has probably been more work done on the study of this disease since our last meeting than on any one subject in medicine. My time is too limited to go into a description of what has been done and the gradual change of opinion that has taken place in the mass of the profession in regard to prognosis.

What I desire to do is to give emphasis to a few points, none of them new, in the diagnosis, prognosis and treatment of these cases as we come in contact with them in our daily work, and to show what can be done, and at the same time keep them practically within the limits of our own State and near their families. The life of these patients in a vast number of cases depends on an early diagnosis, and that is where the greatest error of omission is committed by us daily. We do not give sufficient time and hard work to them. One is often obliged to work three or four weeks on a case and make every known examination before an absolute opinion of the existence or non-existence of the disease can be given.

There is no use taking up the physical signs—they are accessible to us in all text-books. The mass of the profession is not capable, or expected to be capable, of making the examination for the tubercle bacilli, but that is no excuse for not having it done, and repeatedly if necessary; for there is not one of us who is not within reach of a laboratory where it can be done at a small expense. The existence of the bacillus is pathognomonic; its non-existence in the sputa does not exclude disease.

When a patient presents himself with a feeling of malaise, a slight daily variation of temperature, progressive loss of weight, cough as a rule not a very prominent symptom, usually some acceleration of the pulse and loss of appetite, and this condition persists for three or four weeks in spite of the usual line of medication, we are remiss in our duty if we do not diagnose or absolutely exclude phthisis, which in many cases can only be done by keeping

*Read before the Georgia Medical Association, Savannah, April, 1902.

a constant weight and temperature record, repeated, systematic and thorough physical examination combined with examinations of the sputa. The most common errors in my experience are mistaking early phthisis for bronchitis, influenza, typhoid fever, malarial fever and biliousness, whatever that may be.

In 1878 I heard Austin Flint refer to phthisis as an acute disease with an intrinsic tendency towards recovery. He was as usual about twenty years ahead of his time.

With our present method of examination, if the diagnosis is made as soon as it can be, and a proper treatment begun, with a fair cooperation on the part of the patient, there is no reason why the prognosis should be any more grave than that of typhoid fever or pneumonia. Each day that the recognition of the condition is delayed simply adds progressive gravity to the prognosis. Still it is wonderful to see cases recover where the disease was so far advanced as to seem absolutely hopeless.

The three principal factors in the prognosis of any given case are: 1st, period of diagnosis; 2d, personal equation; 3d, financial status.

In the personal equation, besides the usual acceptance of the term, I refer especially to ability of the patient to make a complete change of environment, and not only not suffer depression, but fully enjoy his new duties or pleasures. This, in my opinion, by far exceeds in value the financial ability to obtain anything that may be ordered.

Once the diagnosis is made, give a definite opinion to the patient, or some member of his family, that this is a case of consumption, and that such a line of treatment should be carried out. Do not lull their fears by saying there is a tendency towards consumption, there is a weakness of the lungs, or a slight solidification, and thus delay the day of beginning a rational line of treatment. Let any modification in your outline of treatment that could be carried out be on their responsibility and not yours. Numberless lines of medication of a supposed specific type have been used and vaunted often by high authorities in the treatment of this disease. They become popular for a time, are taken up by the mass of the profession, die out, and new ones spring up. Their number is inestimable. You will find all of the so-called specifics that have given anything like good results have been largely combined with open air and hygiene. In my opinion there is no known medicine that has a specific action in pulmonary tuberculosis; that many of our profession will believe to the contrary in as complex a disease as this is quite natural, when in the far more simple condition of enteric fever we still have our lines of specific treatment, and many will tell us how to abort or curtail the period of a given case.

Do not understand that I do not value to the full extent all lines of medication and hygiene that by building up the patient, assisting digestion and controlling unpleasant symptoms lead to the recovery of many of those cases that would otherwise die. That type of medication is rational and invaluable; but that up to the present anything in the line of or approaching a specific has been discovered—no.

It is not wise to advise a patient to go to what is considered a popular consumptive resort. The constant association with people in all stages of the disease can but be depressing to the most stoical, and one of the greatest elements of cure is hope. It is impossible to have as good hygienic surroundings in a place where phthisical patients congregate as can be obtained elsewhere. I make this statement without fear of contradiction, and do not care what rules may be enforced or what methods of disinfection are employed in any given locality. Think for a moment what must be the history of any room that a stranger can get in an ordinary place that has been a consumptive resort for years.

Since time immemorial a change of climate has been advised in phthisis. Good results have been obtained on the seacoast, in the mountains, below the sea level, in the deserts, in the frigid, temperate and tropical zones. There is only one thing that is common to all these—pure air. That a given case may do better in one temperature or altitude than in another is rational, but with the mass of cases it is simply a question of constant living in the open air; and other minor points aside, the best climate is where the weather permits a given patient to be out of doors the greatest amount of time. The next most important element is occupation. There is nothing more pitiable than the poor invalid who has nothing in the world that interests or amuses him. When you have a case that has absolutely made up his mind to die, and after going over all the outdoor amusements and occupations that are within his reach, you find it impossible to interest him in any of them, it is best for him to stay at home and get what care his family can give him. He will live longer. A vast majority of our cases can do better in the limits of our own State than elsewhere. Few of us realize the diversity of our climate. Within our borders it is a question of a few hours from the trailing arbutus to the yellow jessamine, from the fig to the apple, from the palmetto to the hazel-nut, from the ruffed grouse to the mocking-bird and from the brook-trout to the tarpon.

A varying altitude from the sea to 5,000 feet elevation gives us an all-the-year-round climate and variations in surroundings unequalled in any State east of the Mississippi River, and I have yet to find the case of tuberculosis that from a climatological standpoint cannot be put in proper surroundings by making a few short and easy journeys during the year.

The best treatment for the vast majority of cases that are not absolutely bedridden is unquestionably life in a camp. This gives them the maximum amount of open air, and around the camp there is such a multitude of duties to perform and so many field-sports to be indulged in that it is rare to find a patient that will not be constantly occupied with some congenial task, which can always be regulated according to his strength. Then the very essential element of good and well-cooked food can be better obtained in a camp than elsewhere. Fresh vegetables and fruits in season, with the finest milk, butter, eggs, chickens and mutton all the year around, besides fish and game, furnish a bill of fare that, if properly handled, cannot be excelled anywhere in the world. The amount of food that one of our patients, who could practically eat nothing in the city, will eat and digest after a few days in the woods is astonishing. The cost of camp-life, like everything else, depends on the individual, but everything obtainable for the commissary department can be bought in a camp anywhere in the limits of this State at a cost of from five to ten dollars per capita per month.

One party camped out in Fannin county last year and had a table that was equalled by no hotel in this State at a cost of $5.40 per capita.

In camping, I prefer a log or board house with a good roof and floor to any tent. They are drier in bad weather and the air cannot be so easily excluded. Such houses can be found and obtained at a trifling rental all through the country, or if preferable, can be built at so small a cost that they can be abandoned at the end of the season. Permission to camp, with all field-sports included, can be obtained free of cost by any respectable person anywhere in the State.

Next to camp life, boarding on a good farm can be advised, but as a rule the pleasures and freedom are curtailed, and as well-cooked food cannot be obtained as can be furnished by your own selected cook in camp. There is also more liability to be a lack of occupation.

Next comes the small country hotel with the same drawbacks and a few more. Excellent farm and country hotel board exists, but truly fortunate is he who finds it. I always have a few such places on my list and am constantly looking for others.

I will now report a few cases, and will state that when I refer to these cases as cured, I mean that they have at present no constitutional symptoms—physical signs of tubercle bacilli—and, as far as any examination can carry us, they are all physically perfect. None of them were under professional supervision while in the country, and none of them had practically any medicine.

CASE No. 1. Miss X, fifteen years old. Diagnosis April, 1900, previous maximum weight 98 pounds, at time of diagnosis 82, present 112. Spent two summers in private family in small village in North Georgia, two winters in Macon, but in open air as much as possible.

CASE No. 2. Mrs. A., twenty-eight years old. Diagnosis spring of 1901, weight 108, at present 125. All last summer in small village North Georgia.

CASE No. 3. Miss W., twenty-five years. Diagnosis spring of 1899, weight 120, at present 135. One summer in country hotel Western North Carolina, one in camp and farmhouse North Georgia.

CASE No. 4. Mrs. C., twenty-five years. Diagnosis May, 1899, previous maximum weight 116 pounds, time of diagnosis 105, at present 140. Summers in mountains North Carolina and North Georgia, small villages and farms.

In none of the above cases was the disease in its incipiency, but they should have gotten well, and did.

CASE No. 5. Mr. K. had more bacilli in his sputa than I ever saw in any specimen before, and gave every indication of a case of so-called galloping consumption. He lives in Middle Georgia and could not afford a change. He seined daily the first summer and hunted during the winter; his previous work had been indoors and in dusty surroundings. He has lived outdoors since I first saw him. A copy of his letter tells his story:

MARCH 13, 1902.

DEAR SIR—You first saw me in August, 1900. I am now thirty-six years old. The most I ever weighed prior to my sickness was 158. The day you examined me I weighed 135, fever 101½ and having night-sweats every night. My weight now is 155, and has been for over a year.

Yours truly, K.

CASE No. 6. Mr. B.. Diagnosis April, 1901. Third stage. Could not walk fifty yards without assistance. Case appeared hopeless. Previous maximum weight 145, time of diagnosis 102, present 140. Spent summer and fall in small hotels North Georgia. In early fall was taking daily walks of six miles; has worked on a farm in Middle Georgia in all weathers during the past winters.

CASE No. 7. Mr. T. Diagnosis of cavity in 1877 or '78 by Drs. Flint and Loomis. Carried to Florida on a litter with fatal prognosis. Three years in camp, outdoor life ever since. Weight then 96, in past years from 140 to 160. Carries $50,000 life insurance procured regardless of history at home office of companies.

